# *Chlorella pyrenoidosa* mitigated the negative effect of cylindrospermopsin-producing and non-cylindrospermopsin-producing *Raphidiopsis raciborskii* on *Daphnia magna* as a dietary supplement

**DOI:** 10.3389/fmicb.2023.1292277

**Published:** 2023-11-16

**Authors:** Lamei Lei, Shuyan Lai, Wei Liu, Yaokai Li, Huiping Zhang, Yali Tang

**Affiliations:** Department of Ecology, Jinan University, Guangzhou, China

**Keywords:** *Raphidiopsis raciborskii*, zooplankton, essential fatty acids, feeding experiments, nutritional supplements

## Abstract

Feeding effects are crucial for evaluating the capacity of zooplankton to regulate phytoplankton populations within freshwater ecosystems. To examine the impact of the bloom-forming cyanobacteria *Raphidiopsis raciborskii*, which occurs in tropical and subtropical freshwaters, on the growth of zooplankton *Daphnia* in relation to toxins, filament length and fatty acid content, we fed *D. magna* with *R. raciborskii* only (cylindrospermopsin (CYN)-producing and non-CYN-producing, as the negative controls), *Chlorella pyrenoidosa* only (as the positive control) and a mixed diet containing *R. raciborskii* (CYN-producing and non-CYN-producing) and *C. pyrenoidosa*. Consequently, our findings revealed that the toxic effect of CYN-producing *R. raciborskii* strains on *Daphnia* was mitigated by the coexistence of *C. pyrenoidosa* containing stearidonic acid (SDA, C18:4 ω3) in mixed diets. This was evident in the elevated survival rate compared that from diets containing only *R. raciborskii* and a significantly higher reproduction and population intrinsic increase rate compared to diets consisting of only *R. raciborskii* or *C. pyrenoidos*. Additionally, a strong positive correlation was observed between arachidonic acid (ARA, 20:4ω6) and the population intrinsic increase rate of *Daphnia*; notably, *R. raciborskii* strains were found to be rich in the ω6 polyunsaturated fatty acid ARA. These outcomes reinforce the crucial role of polyunsaturated fatty acids in predicting the population increase of crustacean zooplankton, which has long been neglected. Furthermore, our results underscore the potential effectiveness of zooplankton, particularly in temperate lakes, in controlling CYN-producing *R. raciborskii* populations.

## Introduction

The outbreak of cyanobacterial blooms, a symptom of eutrophication in water bodies, disrupts the balance of aquatic ecosystems. Certain harmful cyanobacteria have the ability to produce hepatotoxins or neurotoxins, as well as other unknown toxic compounds, which have a serious negative impact on the safety of aquatic organisms and human health (Dittmann and Wiegand, 2006; Buratti et al., 2017). *Raphidiopsis raciborskii* (previously known as *Cylindrospermopsis raciborskii*), recognized as one of the most successful bloom-forming cyanobacteria in freshwater, has been described as a tropical species. However, its recent expansion into temperate regions has made it a cosmopolitan species in freshwater systems around the world (Antunes et al., 2015; Wu et al., 2022). Notably, *R. raciborskii* can produce diverse cyanotoxins, including cynlindrospermopsin (CYN) and saxitoxin. Exposure to CYN may result in severe cytotoxicity, genotoxicity, and reproductive toxicity, posing a serious risk to the health of both humans and animals (Buratti et al., 2017).

Zooplankton, encompassing a vital group of primary consumers, play an important role as effective grazers of phytoplankton; nevertheless, they are assumed to be negatively affected by cyanobacterial metabolites (toxicity hypothesis) (Codd, 2000; Ger et al., 2014; Lyu et al., 2016a,b). Initially, this toxicity only referred to microcystins, and currently, the focus has shifted toward other cyanotoxins (Wilson et al., 2006; Schwarzenberger, 2022). Among these, CYN is the most commonly reported compound produced by *R. raciborskii* (Rzymski and Poniedziałek, 2014). Feeding zooplankton with a CYN-producing *R. raciborskii* led to higher mortality and lower growth in *D. magna* juveniles compared to feeding them a non-CYN-producing strain (Nogueira et al., 2004, 2006). Intriguingly, a CYN-producing strain did not exhibit lethal toxicity toward three *Daphnia* species (Hawkins and Lampert, 1989). Due to limited studies, the effects of CYN-producing *R. raciborskii* on *Daphnia* remain elusive (Schwarzenberger, 2022). Toxic effects seem to be strain specific, and different *Daphnia* species display different sensitivities to cyanotoxin exposure (Wilson et al., 2006; Ferrão-Filho et al., 2008; Costa et al., 2013). Additionally, it is noteworthy that zooplankton possess the ability to gradually develop desensitization to toxins through a series of adaptive mechanisms when coexisting with cyanobacteria (Kirk and Gilbert, 1992; Ka et al., 2012; Lyu et al., 2016a), minimizing adverse effects arising from toxin exposure.

The reduced feeding activity of *Daphnia* when fed *R. raciborskii* has been attributed to potential mechanical interference caused by long filaments impeding the feeding apparatus of grazers (Gliwicz and Lampert, 1990; DeMott et al., 2001; Bednarska et al., 2014), hence leading to negative effects on the growth and reproduction of *Daphnia*. However, Rangel et al. (2016) argued that toxicity may override morphology regarding the effects of toxic *R. raciborskii* on zooplankton. Some laboratory experiments even demonstrated that the filament length of *R. raciborskii* did not have a distinct influence on the clearance rates of *D. magna* (Panosso and Lürling, 2010). *D. galeata* actually benefits from the presence of filaments in the food suspension (Abrusán, 2004). Furthermore, by synthesizing data from 66 published laboratory studies, representing 597 experimental comparisons, Wilson et al. (2006) revealed that filamentous cyanobacteria were indeed found to be notably better food sources for grazers than single-celled cyanobacteria across all the studies. Thus, feeding inhibition by filaments may not hold the same level of significance as previously described.

Apart from filament length or toxins, the poor food quality offered by cyanobacteria may also exert adverse effects on the growth and reproduction of zooplankton. iTRAQ-Based proteomic profiling indicated that when exposing to microcystin-producing and microcystin-free *Microcystis aeruginosa*, *Daphnia* showed 94 and 117 differentially expressed proteins respectively, all of which correspond to changes in metabolism necessary to adjust the body growth rate of *Daphnia* (Lyu et al., 2016b). Food quality, including various essential elements and biochemicals, may constrain consumer performance by specifically affecting physiological processes and thus disrupt energy flow in aquatic food webs (Becker and Boersma, 2005; Ruiz et al., 2021). The essential biochemicals cyanobacteria lack but are vital for consumers include polyunsaturated fatty acids (PUFAs), especially eicosapentaenoic acid (EPA, C20: 5ω3, Gulati and DeMott, 1997), or alternative resources, such as an effective EPA enhancing fatty acid, namely, stearidonic acid (SDA, C18:4 ω3, Lenihan-Geels et al., 2013; Abonyi et al., 2023). These PUFAs play a crucial role in maintaining membrane structure and function and serve as precursors for bioactive compounds in both vertebrates and invertebrates, and their *de novo* synthesis is very scarce (Kainz et al., 2009; Twining et al., 2021). In addition, these ω3 fatty acids have been reported to attenuate the toxic effects of various oxidative stresses in mammals (Haimeur et al., 2012; Sakai et al., 2017). The deficiency of EPA in diets restricts the growth of zooplankton (Müller-Navarra, 1995a; Brett et al., 2009; Tang et al., 2019), impacting the performance of crustacean grazers within aquatic ecosystems according to lake investigations and experimental studies (Müller-Navarra et al., 2000; Tang et al., 2023). More importantly, the lack of dietary supply of both EPA and SDA can dramatically affect the reproduction of *Daphnia* due to the high investment of EPA in eggs (Wacker and Martin-Creuzburg, 2007; Kainz et al., 2009). Consequently, cyanobacteria can hardly support the somatic growth and reproduction of zooplankton even in the absence of toxins (Lampert, 1987). To date, no research has revealed the fatty acid profile of *R. raciborskii*. However, we assume that *R. raciborskii* strains, being cyanobacteria, also lack EPA or SDA.

Despite the shortage of EPA or SDA, *R. raciborskii* strains could provide essential nutritional components necessary for zooplankton. These components encompass carbohydrates, proteins, and common saturated and unsaturated fatty acids. It is noted that feeding *Daphnia* with *R. raciborskii* cells only is rarely seen in nature. Concurrently, other phytoplankton species of considerable nutritional value coexist with *R. raciborskii,* even during *Raphidiopsis* blooms (Soares et al., 2009; Chislock et al., 2014; Frau et al., 2018). Given the relatively low EPA need in zooplankton (Müller-Navarra et al., 2000; Becker and Boersma, 2005; Tang et al., 2019). Wenzel et al. (2021) proposed that food sources, such as bacteria, which may not fully meet grazers’ dietary needs, could still confer nutritional benefits if other complementary food components are available in sufficient quantities to compensate for any biochemical deficiencies. Interestingly, even lower-quality food such as vascular plants can be utilized by zooplankton when simultaneously provided with algal food (Taipale et al., 2016; Tang et al., 2021). Considering the role of ω3 fatty acids in detoxication, we hypothesized that the performance of zooplankton feeding *R. raciborskii* would be dramatically improved by the concurrent feeding of good-quality algae. Reis et al. (2023) reported that the fitness of these small-bodied cladocerans feeding on *R. raciborskii* was improved when the supply of nutritious food increased from 10 to 50% in proportion. In natural lakes, the co-occurrence of *R. raciborskii* bloom and filter-feeding zooplankton is commonly seen as previously reported (Bouvy et al., 2001; Leonard and Paerl, 2005; Soares et al., 2009; Gao et al., 2022).

To test our hypothesis, we conducted a feeding experiment to compare the dietary effect of *R. raciborskii* only (CYN- and non-CYN-producing strains), *Chlorella pyrenoidosa* only and a mixed diet of *R. raciborskii* and *C. pyrenoidosa* on the growth, reproduction and population dynamics of *Daphnia magna*. Additionally, we analyzed the fatty acid profile of these different dietary algal strains to uncover the role of specific fatty acids in influencing the growth and reproduction of *Daphnia*. Our findings revealed that the mixed diet led to a higher survival rate of *Daphnia* compared to the *R. raciborskii* only diet and an even higher population intrinsic growth rate compared to the *C. pyrenoidos* only diet. The presence of *C. pyrenoidosa* appears to diminish the toxic effect of CYN-producing *R. raciborskii* strains. In addition, the strongly positive relationship between ω6 PUFA and the population intrinsic increase rate of *Daphnia*, as well as the rich content of ω6 PUFA ARA in *R. raciborskii,* indicates that *R. raciborskii* might be beneficial for the population increase of *Daphnia* as a nutritional supplement. Elevated zooplankton populations to phytoplankton ratios normally indicate a more robust capacity for phytoplankton control (Søndergaard et al., 2008; Jeppesen et al., 2012). Thus, our results point toward the potential for employing a top-down biomanipulation approach to control *R. raciborskii* blooms, particularly in temperate areas.

## Materials and methods

### Experimental algae and animals

The green algae *Chlorella pyrenoidosa* was obtained from the Institute of Hydrobiology, Jinan University. Two CYN-producing strains (*R. raciborskii* CS506 and QDH7) and one non-CYN-producing strain (*R. raciborskii* N8) were used in the experiments. *R. raciborskii* CS506 was obtained from the Australian National Algae Culture Collection (ANACC) and can produce CYN and deoxy-CYN (Willis et al., 2015). Strain QDH7 mainly produced deoxy-CYN, which was identified by LC–MS/MS analysis (Lu et al., 2020). *R. raciborskii* N8 is a nontoxic strain due to the absence of the CYN biosynthesis gene cluster in its whole genome (Chen et al., 2022). All four strains were grown on BG11 medium at 28°C at a light intensity of 60 μmol m^−2^ s^−1^ in a 12:12 h light/dark cycle. Under these conditions, *C. pyrenoidosa* grew as single-cell populations with an average diameter of 4.1 μm. Filaments of *R. raciborskii* N8, QDH7 and CS506 had average lengths of 387 μm, 902 μm and 1,214 μm, respectively (Table 1).

**Table 1:** General characteristics of the algal strains used in the study.

Species	Strain	Origin	Toxin type	Mean filament length (μm)
*R. raciborskii*	N8	China	Nontoxic	387
	QDH7	China	Deoxy-CYN (19.8 μg mg^−1^ dry weight)	903
	CS506	Australia	CYN, deoxy-CYN (14.4 μg mg^−1^ dry weight)	1,214
*C. pyrenoidosa*	CP	China	Nontoxic	4.1

The cladoceran *D. magna* was maintained at 20°C and fed with the green algae *C. pyrenoidosa* in 1-L glass jars. Water from Liuxihe Reservoir in Guangzhu city was used to prepare all media after sequential filtration through a 1.2 and 0.45-μm filter. The filtrate was stored statically at 25°C for 2 days before use. Neonates (<24 h old) were randomly chosen from parthenogenetically reproducing females for the life history experiments.

### Feeding experiments

*Daphnia magna* was fed three different kinds of diets: *C. pyrenoidosa* only, 1:1 mixtures of *C. pyrenoidosa* with either *R. raciborskii* N8, QDH7 or CS506, and *R. raciborskii* (N8, QDH7 or CS506) only. All diets had a fixed total food concentration of 2 mg C L^−1^. The food carbon concentrations were determined using OD (optical extinction) values according to the regression curve we built for OD and carbon concentration for different algae at 682 nm.

Before the life history experiments, neonates (<24 h old) originating from the same broods were collected from beakers and starved for 4 h to empty their guts. Thirty neonates were randomly selected for each treatment. Each neonate was transferred into 50 mL of food suspension and incubated under the same conditions as the stock *D. magna* cultures. Each selected neonate was transferred daily to clean beakers with freshly prepared food suspensions. Any observed offspring were removed immediately after their presence was recorded. The experiments lasted for 15 days.

The body lengths of zooplankton were measured using a stereo binocular microscope, and the biomass was calculated based on the body length measurement. Somatic growth rates at the juvenile stage (g) were determined by assessing the increase in biomass from Day 1 (M_1_) to Day 7 (M_7_) within the experimental period (*t* = 6 d) using the Equation g = (ln M_7_ – ln M_1_)/t. In addition, the survival rate and the daily number of offspring produced were also recorded.

The intrinsic rates of increase r (d^−1^) in the population of *D. magna* were calculated using Euler’s formula as follows:


$$1=\overset{n}{\underset{x=0}{\sum }}e^{-rx}\times l_{x}\times m_{x}$$

where *x* is the age or time interval (day), *l_x_* is the proportion of individuals surviving to age *x*, and *m_x_* represents the number of offspring produced per surviving female at age *x*.

### Fatty acid analysis and CYN measurement

The fatty acids within the cells of *C. pyrenoidosa* and three *R. raciborskii* strains were extracted and esterified according to the method outlined by Wiltshire et al. (2000). Quantitative analysis was carried out using a gas chromatography–mass spectrometer (GC–MS) with a specific temperature configuration. The cultures of the four strains were collected by centrifugation and then subjected to freeze-drying. Approximately 20 mg of dry biomass was utilized to extract total lipids three times with dichloromethane/methanol (2:1, v/v), and the pooled cell-free extracts were evaporated to dryness. Subsequently, the extracted samples were transesterified with 3 mol L^−1^ methanolic HCl (60°C, 15 min). Fatty acids were analyzed with a Finnigan TRACE GC–MS equipped with a flame ionization detector and a DB-23 column (60 m × 0.32 mm). The fatty acid methyl esters (FAME) were quantified by comparison with standard Supelco 37 Component FAME mix or an internal standard (C12:0 methyl esters).

Total CYN concentrations in *R. raciborskii* QDH7 and CS506 cells were measured before the feeding experiments. Prior to CYN measurement, *R. raciborskii* cells were lysed by ultrasonic treatment, and insoluble cell debris was removed by centrifugation. The supernatant was then analyzed with a Cylindrospermopsin Plate Kit (Beacon Analytical Systems Inc., USA) in accordance with the manufacturer’s specifications.

### Data analysis

To assess and compare the fatty acid (FA) content across different diet treatments, a one-way analysis of variance (ANOVA) was employed. Significant differences among treatments were then evaluated using the least significant difference (LSD) multiple comparison test. One-way ANOVA followed by the LSD test was also performed to identify differences in the growth and reproduction of *D. magna* between different diets. Regression analyses were performed to determine the relationship between population intrinsic increase rates and PUFA content in diets. An SPSS 22.0 statistical package was used for all statistical analyses. Before the statistical analysis, data were checked using a normal probability plot of the residuals and Levene’s test of homogeneity of variances for compliance of ANOVA assumptions and logarithmic transformation, if necessary. Detailed information on the statistics is presented in the Supplementary Table S1.

## Results

### Fatty acid composition

**Figure 1:**
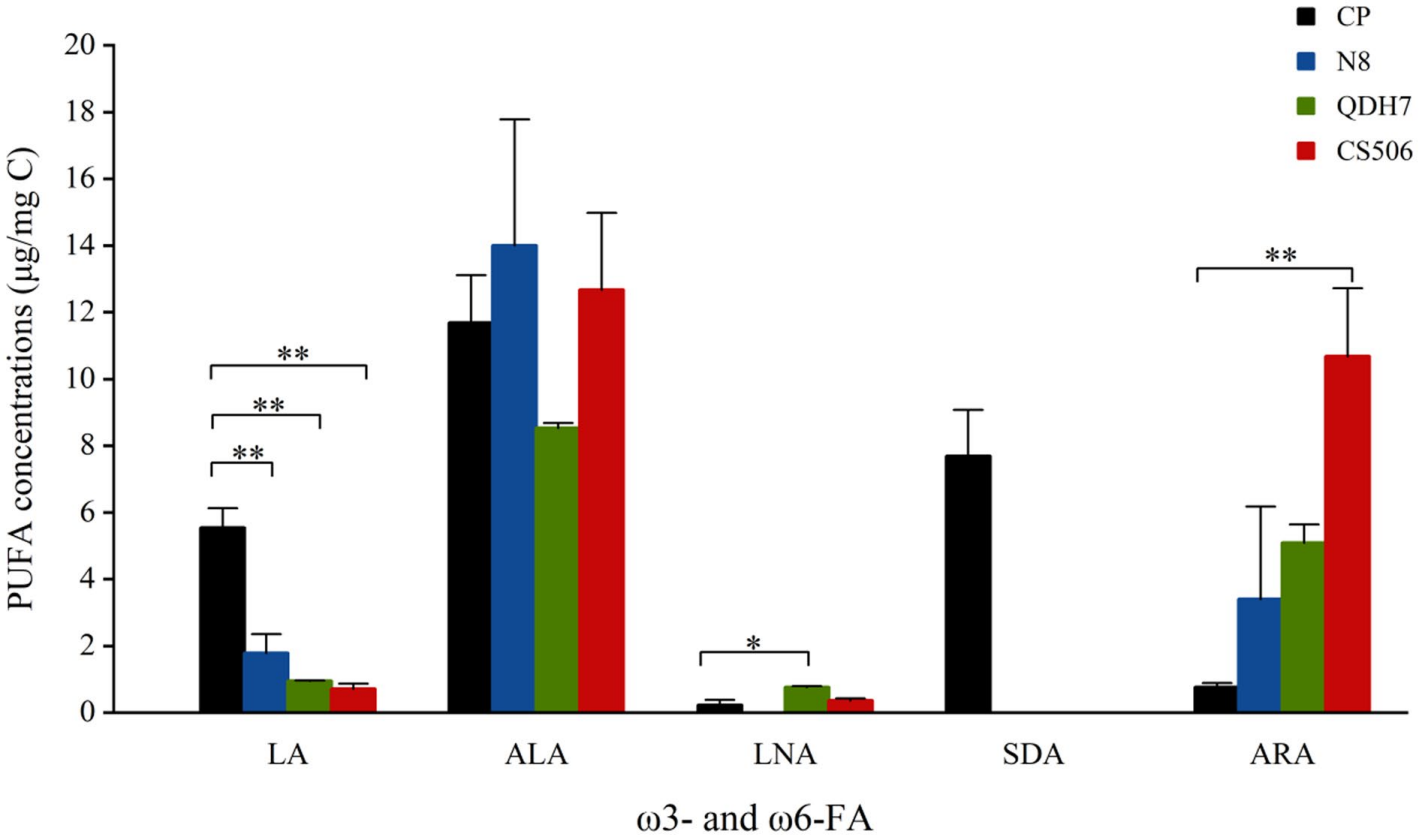
The main PUFA concentrations (μg/mg C) of *C. pyrenoidosa* (CP) and three strains of *R. raciborskii* (N8, QDH7 and CS506). Error bars indicate 1SD. The “*” above the bars are significantly different from CP, as revealed by the independent-samples *t* test (*p < 0.05; **p < 0.01).

The green algae *C. pyrenoidosa* was characterized by high amounts of C16:0 and C18:0 saturated fatty acids, very small amounts of monounsaturated fatty acids and considerable amounts of PUFAs, mainly including C18:2ω6 (linoleic acid, LA), C18:3ω3 (linolenic acid, ALA) and C18:4ω3 (stearidonic acid, SDA) (Supplementary Table S1). All *R. raciborskii* strains lacked SDA but exhibited significantly higher levels of C20:4ω6 (arachidonic acid, ARA) than *C. pyrenoidosa* (Figure 1). Detailed information on the fatty acid profile of the algae is presented in the Supplementary Table S2.

### Dietary treatment effects on the performance of *Daphnia* individuals

**Figure 2:**
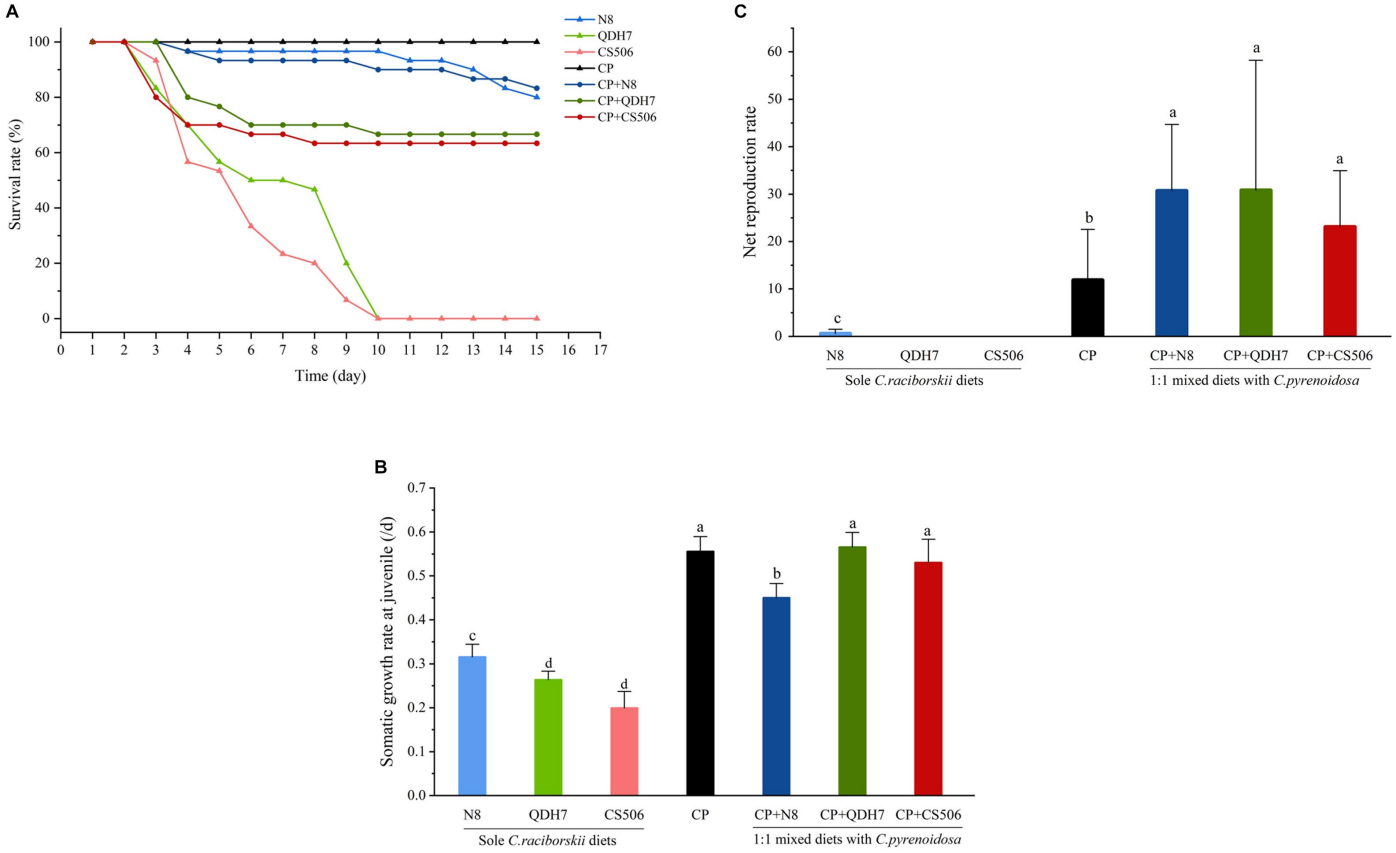
Individual performance of *D. magna* fed with pure diets of *C. pyrenoidosa*, three strains of *R. raciborskii* and 1:1 mixed diets with *C. pyrenoidosa* and *R. raciborskii* strains including survival rate **(A)** somatic growth rate at juvenile **(B)** and net reproduction rate if reproduced after juvenile **(C)**. Different letters indicate significant differences (*p* < 0.05) using the LSD multiple comparison test.

In the case of pure *R. raciborskii* dietary treatments, all *D. magna* exposed to 100% QDH7 and 100% CS506 strains experienced toxicity, resulting in mortality within 9 days. When *C. pyrenoidosa* was added to the diets with two toxic *R. raciborskii* strains, the survival rate was greatly elevated to above 60% (Figure 2A).

Compared with the diet comprising only *C. pyrenoidosa*, the growth rates of *D. magna* fed only *R. raciborskii* were significantly lower (Figure 2B). *D. magna* exhibited better growth when fed pure diets of non-CYN-producing N8 compared to the CYN-producing strains (QDH7 and CS506). In terms of inhibiting *D. magna* growth, no significant difference was observed between the aforementioned two toxic strains. Interestingly, *R. raciborskii* supplemented with *C. pyrenoidosa* enhanced the growth of *D. magna. Daphnia* fed mixed diets displayed significantly better growth than those exclusively fed *R. raciborskii*, similar to the positive control group that was fed *C. pyrenoidosa*.

In the pure *R. raciborskii* dietary treatments, all *D. magna* exposed to 100% QDH7 and 100% CS506 strains did not reproduce. The *D. magna* on the pure diets of the non-CYN-producing N8 strain was able to reproduce successfully but showed the lowest net reproduction value. When all *Daphnia* were fed mixed diets, they exhibited robust net reproduction rates comparable to the positive control group (Figure 2C). The maximum net reproduction (net value) was observed in the 50% QDH7 treatment.

### Intrinsic population increase rate of *Daphnia* and its relation with dietary PUFA supply

**Figure 3:**
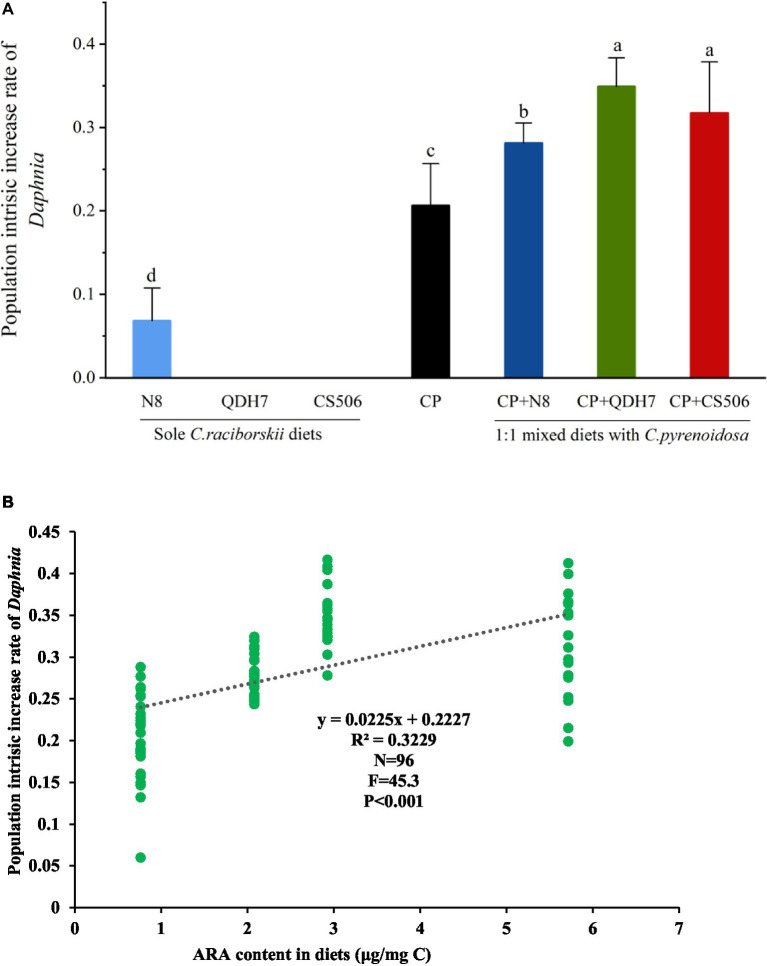
The intrinsic rate of population increase of *D. magna* fed with pure diets of *C. pyrenoidosa*, three strains of *R. raciborskii* and 1:1 mixed diets with *C. pyrenoidosa* and *R. raciborskii* strains if they reproduced **(A)** and its relation with dietary supply of arachidonic acid (ARA, 20:4 ω6) within stearidonic acid (SDA, 18:4 ω3)-containing diets **(B)**. Different letters indicate significant differences (*p* < 0.05) using the LSD multiple comparison test.

The intrinsic rate of population increase of *Daphnia* feeding mixed diets was found to be significantly higher than that of the positive control group feeding *C. pyrenoidosa* (Figure 3A). Among all the mixed dietary treatments, the maximal population intrinsic increase rate (mean value) was found in the 50% QDH7 treatment, and the lowest was found in the 50% N8 treatment. Since *D. magna* exposed to 100% QDH7 and 100% CS506 strains showed no reproduction, their population intrinsic increase rate could not be calculated. Regression analyses revealed a very strong correlation between the ARA content in diets and the population intrinsic increase rates of *Daphnia* in all dietary treatments containing SDA (Figure 3B).

## Discussion

By setting up feeding experiments to compare the dietary impact of different food sources on the growth and reproduction of *Daphnia*, we found that the highest intrinsic population growth rate was observed in *Daphnia* fed a mixed diet, followed by those on a diet of *C. pyrenoidosa*, then non-CYN-producing *R. raciborskii*, and finally, the lowest growth rate was seen in *Daphnia* fed with CYN-producing *R. raciborskii*. Thus, a mitigation of the negative effect of CYN-producing and non-CYN-producing *R. raciborskii* on *D. magna* by *C. pyrenoidosa* as diet supplements was observed, inconsistent with our hypothesis.

In our study, the ability to produce CYN had a notable impact on the growth of *Daphnia*. Among the treatments involving a sole diet, *Daphnia* fed on two toxic strains exhibited significantly reduced growth and reproduction rates compared to those fed on nontoxic strain N8 or *C. pyrenoidosa*, consistent with the observation made by Nogueira et al. (2004, 2006), underscoring the detrimental effect of CYN toxins. Strikingly, the negative effects of the toxins produced by both toxic *R. raciborskii* strains on *Daphnia* were mitigated when *C. pyrenoidosa* was concurrently provided as part of their diets, particularly in terms of the intrinsic population increase rate.

Based on the fatty acid profiles of dietary algae, we further proposed that the differences in *Daphnia* performance across various dietary treatments can be attributed to variations in the fatty acid contents. One particularly crucial fatty acid, EPA, has been shown to limit *Daphnia* growth and reproduction both in laboratory settings and in natural environments, and it also plays a predictive role in carbon transfer between primary producers and consumers (Müller-Navarra, 1995b; Müller-Navarra et al., 2000). Both SDA and ALA can be converted to EPA by consumers (Kainz et al., 2009). Due to the low conversion rate of ALA to EPA, researchers have noted that supplying SDA may increase EPA levels more effectively than ALA supplementation by bypassing a rate-limiting step (Lenihan-Geels et al., 2013; Abonyi et al., 2023). The *R. raciborskii* strains lack both the crucial ω3 PUFA EPA and the alternative EPA precursor SDA. This deficiency in essential fatty acids contributes to the poor food quality of *R. raciborskii* and significantly limited the population increase of *Daphnia* in our study. Even when fed its non-CNY-producing strain, *Daphnia* showed considerably lower growth and reproduction rates than those fed *C. pyrenoidosa*. It was evident that food containing SDA (including mixed diets and *C. pyrenoidosa* only diet) significantly supported growth and reproduction when compared to food lacking SDA (such as different strains of *R. raciborskii* as only diets).

More importantly, it is worth noting that oxidative stress, induced by the rapid increase in the production of reactive oxygen species (ROS), represents a pivotal mechanism underlying CYN toxicity (Poniedzialek et al., 2015). EPA has been documented to attenuate oxidative stress-induced DNA damage and elevate glutathione peroxidase activity in mammals (Haimeur et al., 2012; Sakai et al., 2017). Moreover, increased levels of glutathione peroxidase have been demonstrated to participate in the detoxication of cyanotoxins, including CYN, in *Daphnia* (Nogueira et al., 2004; Lindsay et al., 2006; Schwarzenberger, 2022). Hence, it is plausible that the SDA derived from the supplemented *C. pyrenoidosa* enhanced the EPA content in zooplankton, eventually mitigating the adverse effects of toxic *R. raciborskii* strains on *Daphnia magna*. The mitigation of more dietary chlorophyte addition on the performance of *Daphnia* fed toxic *R. raciborskii* strains could also be demonstrated by data from Reis et al. (2023) for growth and reproduction, as well as Panosso and Lürling (2010) for feeding rate, supporting our view.

Unlike *Microcystis* (Ahlgren et al., 1992), *R. raciborskii* strains in our study were rich in ω6 PUFA ARA. Although most ecologists pay more attention to ω3 PUFA (Twining et al., 2021), inadequate availability of ω6 PUFA ARA can also constrain the fitness of *Daphnia* (Ilić et al., 2019), especially when EPA or EPA-enhancing fatty acid SDA is already present in their diet. ARA serves as a precursor for tissue hormones such as prostaglandin and related eicosanoids, which play critical roles in mediating reproduction (Heckmann et al., 2008; Stanley and Kim, 2019). Previously, a ω3/ω6 ratio ranging from 2.6 to 4.0 was reported for wild filtering cladoceran species (Persson and Vrede, 2006). In our study, the observed positive effects of adding *R. raciborskii* on reproduction and population increase might be attributed to nutritional supplements of ω6 PUFA. This speculation could be further demonstrated by the positive correlation between ARA content in the food and the intrinsic population increase rate of *Daphnia* when fed diets containing SDA. Other nutritional components, for example, proteins, may also work here, but we did not determine all the nutritional profiles and focused on essential fatty acids.

The observed beneficial effects of our CYN-producing and non-CYN-producing *R. raciborskii* strain in addition to *C. pyrenoidosa* as diets on the population increase of *D. magna*, however, were different from what Reis et al. (2023) observed. They reported that *R. raciborskii* constrains the fitness of *Daphnia* when this strain was added to chlorophyte as diets, by using different *R. raciborskii* strains (saxitoxin-producing strain), different *Daphnia* species (*D. laveis* and *D. gessneri*) and different chloryphyte species (*Monoraphidium capricornutum* and *Ankistrodesmus stiptatus*). A possible explanation is that algae may exhibit strain differences in their fatty acid profiles (Dunstan et al., 1993). And the fatty acid profile would eventually affected the growth of *Daphnia* if their needs for essential fatty acids were not met (Gulati and Demott, 1997). In addition, different *Daphnia* species, or even clones, may respond differently to different diets according to their different sensitivities to different or the same toxins (Ferrão-Filho et al., 2008; Costa et al., 2013) and nutritional requirements (Ferrão-Filho et al., 2019).

The poor manageability of filamentous *R. raciborskii* previously caused a reduction in both the growth rate and fecundity of *Daphnia* (Bednarska et al., 2014). In our feeding experiments, we observed that the trichomes of strain CS506 were three times longer than those of the N8 strain. Surprisingly, the length of *R. raciborskii* filaments did not appear to have a pronounced impact on *Daphnia* fitness in our study. This finding aligns with the work of Panosso and Lürling (2010), who demonstrated that longer *R. raciborskii* filaments may not necessarily cause stronger feeding inhibition than shorter ones for large-bodied *D. magna* (2–3 mm) within the range they tested. Increasing feeding inhibition in larger body-sized animals exposed to filamentous cyanobacteria were reported (Demott et al., 2001), but the conclusion was not generally-accepted for cladocerans due to their species-specific or clone-specific sensitivities when exposed to cyanobacteria (Bednarska et al., 2014). Relatively high feeding rates of *R. raciborskii* were also reported in daphnids of different body sizes, e.g., 1.1 mm *D. longispina* and 1.4 mm *D. pulicaria*, 1.6 mm *D. laevis* and 2.5 mm *D. similis* (Ferrão-Filho et al., 2017; Sikora and Dawidowicz, 2017; Ferrão-Filho et al., 2020), despite feeding inhibition being previously observed in 0.6–1.3 mm *D. galeata*, 1.2 mm *D. cucullata*, 1.9 mm *D. hyalina*, and 2.3 mm *D. pulicaria* (Schoenberg and Carlson, 1984; Gliwicz and Lampert, 1990). Notably, the *D. magna* clone used in our study might be less sensitive to clogging, as previously described (Soares et al., 2009).

*Daphnia magna* is a typical filter-feeding water flea and has long been used as a model for food quality and aquatic ecotoxicity studies. Most aquatic filter-feeders including cladocerans and rotifers, shared necessary requirements for polyunsaturated fatty acids such as SDA, EPA and ARA etc. (Schälicke et al., 2019; Thomas et al., 2022). Thus, our results lend further support to the idea that crustacean zooplankton may have the potential to control CYN-producing *R. raciborskii* populations in temperate lakes, as previously proposed (Gao et al., 2022), but with a particular emphasis on the nutritional perspective. Compared to temperate lakes, weak top-down effects were recorded due to the presence of fish species that spawn multiple times per year, resulting in an abundance of young-of-the-year fish all year around that prey on the large-bodied zooplankton in tropical and subtropical lakes (Liu et al., 2018; He et al., 2022), decreasing the control of large-bodied zooplankton on large-bodied zooplankton. Ferrão-Filho et al. (2020) showed the control of small-bodied zooplankton by saxitoxin-producing *R. raciborskii* in a mesocosm study; however, they suggested that high nutrient recycling other than the grazing effect by fish might weaken zooplankton’s control on cyanobacteria in trophic areas. Taken together, these findings may partially explain why *R. raciborskii* is more prevalent in tropical and subtropical areas than in temperate areas.

## Conclusion

In summary, *Chlorella pyrenoidosa* relieved the negative effect of cylindrospermopsin-producing and non-cylindrospermopsin-producing *Raphidiopsis raciborskii* on *D. magna* in our study. The findings underscore the potential effectiveness of zooplankton, particularly in temperate lakes, in controlling CYN-producing *R. raciborskii* populations.

## Data availability statement

The original contributions presented in the study are included in the article/Supplementary material, further inquiries can be directed to the corresponding author.

## Author contributions

LL: Data curation, Writing – original draft, Writing – review & editing. SL: Data curation, Writing – review & editing. WL: Methodology, Writing – review & editing. YL: Investigation, Writing – review & editing. HZ: Formal analysis, Writing – review & editing. YT: Conceptualization, Writing – original draft, Writing – review & editing.

## References

[ref1] Abonyi A. Rasconi S. Ptacnik R. Pilecky M. Kainz M. J. (2023). Chytrids enhance Daphnia fitness by selectively retained chytrid-synthesised stearidonic acid and conversion of short-chain to long-chain polyunsaturated fatty acids. Freshw. Biol. 68, 77–90. doi: 10.1111/FWB.14010, PMID: 37064759 PMC10099718

[ref2] Abrusán G. (2004). Filamentous cyanobacteria, temperature and *Daphnia* growth: the role of fluid mechanics. Oecologia 141, 395–401. doi: 10.1007/s00442-004-1660-x, PMID: 15375688

[ref3] Ahlgren G. Gustafsson I. B. Boberg M. (1992). Fatty acid content and chemical composition of freshwater microalgae 1. J. Phycol. 28, 37–50. doi: 10.1111/j.0022-3646.1992.00037.x

[ref4] Antunes J. T. Leão P. N. Vasconcelos V. M. (2015). *Cylindrospermopsis raciborskii*: review of the distribution, phylogeography, and ecophysiology of a global invasive species. Front. Microbiol. 6:473. doi: 10.3389/fmicb.2015.00473, PMID: 26042108 PMC4435233

[ref5] Becker C. Boersma M. (2005). Differential effects of phosphorus and fatty acids on *Daphnia magna* growth and reproduction. Limnol. Oceanogr. 50, 388–397. doi: 10.2307/3597910

[ref6] Bednarska A. Pietrzak B. Pijanowska J. (2014). Effect of poor manageability and low nutritional value of cyanobacteria on *Daphnia magna* life history performance. J. Plankton Res. 36, 838–847. doi: 10.1093/plankt/fbu009

[ref7] Bouvy M. Pagano M. Troussellier M. (2001). Effects of a cyanobacterial bloom (*Cylindrospermopsis raciborskii*) on bacteria and zooplankton communities in Ingazeira reservoir (Northeast Brazil). Aquat. Microb. Ecol. 25, 215–227. doi: 10.3354/ame025215

[ref8] Brett M. T. Kainz M. J. Taipale S. J. Seshan H. (2009). Phytoplankton, not allochthonous carbon, sustains herbivorous zooplankton production. Proc. Natl. Acad. Sci. 106, 21197–21201. doi: 10.1073/pnas.0904129106, PMID: 19934044 PMC2795543

[ref9] Buratti F. M. Manganelli M. Vichi S. Stefanelli M. Scardala S. Testai E.. (2017). Cyanotoxins: producing organisms, occurrence, toxicity, mechanism of action and human health toxicological risk evaluation. Arch. Toxicol. 91, 1049–1130. doi: 10.1007/s00204-016-1913-6, PMID: 28110405

[ref10] Chen Z. J. Ruan Z. X. Cheng N. Xiao L. J. Peng L. Han B.-P.. (2022). Whole-genome sequencing and phosphorus uptake and transport pathway comparative analysis of *Cylindrospermopsis raciborskii* N8. Acta Hydrobiol. Sin. 46, 1130–1141. doi: 10.7541/2022.2021.0197

[ref11] Chislock M. F. Sharp K. L. Wilson A. E. (2014). *Cylindrospermopsis raciborskii* dominates under very low and high nitrogen-to-phosphorus ratios. Water Res. 49, 207–214. doi: 10.1016/j.watres.2013.11.022, PMID: 24333522

[ref12] Codd G. A. (2000). Cyanobacterial toxins, the perception of water quality, and the prioritisation of eutrophication control. Ecol. Eng. 16, 51–60. doi: 10.1016/S0925-8574(00)00089-6

[ref13] Costa S. M. Ferrão-Filho A. S. Azevedo S. M. F. O. (2013). Effects of saxitoxin- and non-saxitoxin-producing strains of the cyanobacterium *Cylindrospermopsis raciborskii* on the fitness of temperate and tropical cladocerans. Harmful Algae 28, 55–63. doi: 10.1016/j.hal.2013.05.017

[ref14] DeMott W. R. Gulati R. D. Donk E. V. (2001). Effects of dietary phosphorus deficiency on the abundance, phosphorus balance, and growth of *Daphnia cucullata* in three hypereutrophic Dutch lakes. Limnol. Oceanogr. 46, 1871–1880. doi: 10.2307/3069058

[ref15] Dittmann E. Wiegand C. (2006). Cyanobacterial toxins–occurrence, biosynthesis and impact on human affairs. Mol. Nutr. Food Res. 50, 7–17. doi: 10.1002/mnfr.200500162, PMID: 16304634

[ref16] Dunstan G. A. Volkman J. K. Barrett S. M. Garland C. D. (1993). Changes in the lipid composition and maximisation of the polyunsaturated fatty acid content of three microalgae grown in mass culture. J. Appl. Phycol. 5, 71–83. doi: 10.1007/BF02182424

[ref17] Ferrão-Filho A. S. Abreu S. S. D. Oliveira T. Magalhães V. F. Pflugmacher S. Silva E. M. (2017). Single and combined effects of microcystin and saxitoxin producing cyanobacteria on the fitness and antioxidant defenses of cladocerans. Environ. Toxicol. Chem. 36, 2689–2697. doi: 10.1002/etc.3819, PMID: 28409869

[ref18] Ferrão-Filho A. D. S. da Costa S. M. Ribeiro M. G. L. Azevedo S. M. (2008). Effects of a saxitoxin-producer strain of *Cylindrospermopsis raciborskii* (cyanobacteria) on the swimming movements of cladocerans. Environ. Toxicol. Int. J. 23, 161–168. doi: 10.1002/tox.20320, PMID: 18214915

[ref19] Ferrão-Filho A. D. S. Dias T. M. Pereira U. J. Dos Santos J. A. A. Kozlowsky-Suzuki B. (2019). Nutritional and toxicity constraints of phytoplankton from a Brazilian reservoir to the fitness of cladoceran species. Environ. Sci. Pollut. Res. 26, 12881–12893. doi: 10.1007/s11356-019-04851-6, PMID: 30887454

[ref20] Ferrão-Filho A. S. Pereira U. J. Vilar M. C. de Magalhães L. Marinho M. M. (2020). Can small-bodied *Daphnia* control *Raphidiopsis raciborskii* in eutrophic tropical lakes? A mesocosm experiment. Environ. Sci. Pollut. Res. 27, 35459–35473. doi: 10.1007/s11356-020-09737-6, PMID: 32592062

[ref21] Frau D. de Tezanos Pinto P. Mayora G. (2018). Are cyanobacteria total, specific and trait abundance regulated by the same environmental variables? Ann. Limnol. - Int. J. Lim. 54:3. doi: 10.1051/limn/2017030

[ref22] Gao X. Wang W. Ndayishimiye J. C. Govaert L. Chen H. Jeppesen E.. (2022). Invasive and toxic cyanobacteria regulate allochthonous resource use and community niche width of reservoir zooplankton. Freshw. Biol. 67, 1344–1356. doi: 10.1111/FWB.13921

[ref23] Ger K. A. Hansson L. A. Lürling M. (2014). Understanding cyanobacteria-zooplankton interactions in a more eutrophic world. Freshw. Biol. 59, 1783–1798. doi: 10.1111/fwb.12393

[ref24] Gliwicz Z. M. Lampert W. (1990). Food thresholds in *Daphnia* species in the absence and presence of blue-green filaments. Ecology 71, 691–702. doi: 10.2307/1940323

[ref25] Gulati R. Demott W. (1997). The role of food quality for zooplankton: remarks on the state-of-the-art, perspectives and priorities. Freshw. Biol. 38, 753–768. doi: 10.1046/j.1365-2427.1997.00275.x

[ref26] Haimeur A. Ulmann L. Mimouni V. Guéno F. Pineau-Vincent F. Meskini N.. (2012). The role of *Odontella aurita*, a marine diatom rich in EPA, as a dietary supplement in dyslipidemia, platelet function and oxidative stress in high-fat fed rats. Lipids Health Dis. 11, 1–13. doi: 10.1186/1476-511X-11-147, PMID: 23110391 PMC3543224

[ref27] Hawkins P. R. Lampert W. (1989). The effect of *Daphnia* body size on fifiltering rate inhibition in the presence of a fifilamentous cyanobacterium. Limnol. Oceanogr. 34, 1084–1089. doi: 10.2307/2837197

[ref28] He H. Qian T. Shen R. Yu J. Li K. Liu Z.. (2022). Piscivore stocking significantly suppresses small fish but does not facilitate a clear-water state in subtropical shallow mesocosms: a biomanipulation experiment. Sci. Total Environ. 842:156967. doi: 10.1016/J.SCITOTENV.2022.156967, PMID: 35764152

[ref29] Heckmann L. H. Sibly R. M. Timmermans M. J. Callaghan A. (2008). Outlining eicosanoid biosynthesis in the crustacean Daphnia. Front. Zool. 5, 11–19. doi: 10.1186/1742-9994-5-11, PMID: 18625039 PMC2483973

[ref30] Ilić M. Werner C. Fink P. (2019). Equal relevance of omega-3 and omega-6 polyunsaturated fatty acids for the fitness of *Daphnia* spp. Limnol. Oceanogr. 64, 2512–2525. doi: 10.1002/lno.11201

[ref31] Jeppesen E. Søndergaard M. Lauridsen T. L. Davidson T. A. Liu Z. Mazzeo N.. (2012). Biomanipulation as a restoration tool to combat eutrophication: recent advances and future challenges. Adv. Ecol. Res. 47, 411–488. doi: 10.1016/B978-0-12-398315-2.00006-5

[ref32] Ka S. Mendoza-Vera J. M. Bouvy M. Champalbert G. N’Gom-Kâ R. Pagano M. (2012). Can tropical freshwater zooplankton graze efficiently on cyanobacteria? Hydrobiologia 679, 119–138. doi: 10.1007/s10750-011-0860-8

[ref33] Kainz M. Brett M. T. Arts M. T. (2009). Lipids in aquatic ecosystems. Springer-Verlag: New York.

[ref34] Kirk K. L. Gilbert J. J. (1992). Variation in herbivore response to chemical defenses -zooplankton foraging on toxic cyanobacteria. Ecology 73, 2208–2217. doi: 10.2307/1941468

[ref35] Lampert W. (1987). Laboratory studies on zooplankton-cyanobacteria interactions. N. Z. J. Mar. Freshw. Res. 21, 483–490. doi: 10.1080/00288330.1987.9516244

[ref36] Lenihan-Geels G. Bishop K. S. Ferguson L. R. (2013). Alternative sources of omega-3 fats: can we find a sustainable substitute for fish? Nutrients 5, 1301–1315. doi: 10.3390/nu5041301, PMID: 23598439 PMC3705349

[ref37] Leonard J. A. Paerl H. W. (2005). Zooplankton community structure, microzooplankton grazing impact, and seston energy content in the St. Johns river system, Florida as influenced by the toxic cyanobacterium *Cylindrospermopsis raciborskii*. Hydrobiologia 537, 89–97. doi: 10.1007/s10750-004-2483-9

[ref38] Lindsay J. Metcalf J. S. Codd G. A. (2006). Protection against the toxicity of microcystin-LR and cylindrospermopsin in *Artemia salina* and *Daphnia* spp. by pre-treatment with cyanobacterial lipopolysaccharide (LPS). Toxicon 48, 995–1001. doi: 10.1016/j.toxicon.2006.07.036, PMID: 16982077

[ref39] Liu Z. Hu J. Zhong P. Zhang X. Ning J. Larsen S. E.. (2018). Successful restoration of a tropical shallow eutrophic lake: strong bottom-up but weak top-down effects recorded. Water Res. 146, 88–97. doi: 10.1016/j.watres.2018.09.007, PMID: 30236468

[ref40] Lu Y. Lei M. Ye J. Lei L. M. Han B.-P. (2020). Intraspecific variation of morphological traits and toxin-producing capacity and phylogenetic analysis for *Cylindrospermopsis raciborskii* from Qiandenghu Lake, Guangdong Province. J. Lake Sci. 32, 144–153. doi: 10.18307/2020.0114

[ref41] Lyu K. Guan H. Wu C. Wang X. Wilson A. E. Yang Z. (2016a). Maternal consumption of non-toxic *Microcystis*, by *Daphnia magna* induces tolerance to toxic *Microcystis* in offspring. Freshw. Biol. 61, 219–228. doi: 10.1111/fwb.12695

[ref42] Lyu K. Meng Q. Zhu X. Dai D. Zhang L. Huang Y.. (2016b). Changes in iTRAQ-based proteomic profiling of the cladoceran *Daphnia magna* exposed to microcystin-producing and microcystin-free *Microcystis aeruginosa*. Environ. Sci. Technol. 50, 4798–4807. doi: 10.1021/acs.est.6b00101, PMID: 27057760

[ref43] Müller-Navarra D. C. (1995a). Evidence that a highly unsaturated fatty acid limits Daphnia growth in nature. Arch. Hydrobiol. 132, 297–307. doi: 10.1127/ARCHIV-HYDROBIOL/132/1995/297

[ref44] Müller-Navarra D. C. (1995b). Biochemical versus mineral limitation in *Daphnia*. Limnol. Oceanogr. 40, 1209–1214. doi: 10.2307/2838677

[ref45] Müller-Navarra D. C. Brett M. T. Liston A. M. Goldman C. R. (2000). A highly unsaturated fatty acid predicts carbon transfer between primary producers and consumers. Nature 403, 74–77. doi: 10.1038/47469, PMID: 10638754

[ref46] Nogueira I. C. Lobo-da-Cunha A. Vasconcelos V. M. (2006). Effects of *Cylindrospermopsis raciborskii* and *Aphanizomenon ovalisporum* (cyanobacteria) ingestion on *Daphnia magna* midgut and associated diverticula epithelium. Aquat. Toxicol. 80, 194–203. doi: 10.1016/j.aquatox.2006.08.008, PMID: 17010452

[ref47] Nogueira I. C. Saker M. L. Pflugmacher S. Wiegand C. Vasconcelos V. M. (2004). Toxicity of the cyanobacterium *Cylindrospermopsis raciborskii* to *Daphnia magna*. Environ. Toxicol. Int. J. 19, 453–459. doi: 10.1002/tox.20050, PMID: 15352261

[ref48] Panosso R. Lürling M. (2010). *Daphnia magna* feeding on *Cylindrospermopsis raciborskii*: the role of food composition, filament length and body size. J. Plankton Res. 32, 1393–1404. doi: 10.1093/plankt/fbq057

[ref49] Persson J. Vrede T. (2006). Polyunsaturated fatty acids in zooplankton: variation due to taxonomy and trophic position. Freshw. Biol. 51, 887–900. doi: 10.1111/j.1365-2427.2006.01540.x

[ref51] Poniedzialek B. Rzymski P. Karczewski J. (2015). The role of the enzymatic antioxidant system in cylindrospermopsin-induced toxicity in human lymphocytes. Toxicol. In Vitro 29, 926–932. doi: 10.1016/j.tiv.2015.03.023, PMID: 25863213

[ref52] Rangel L. M. Ger K. A. Silva L. H. S. Soares M. C. S. Faassen E. J. Lürling M. (2016). Toxicity overrides morphology on *Cylindrospermopsis raciborskii* grazing resistance to the Calanoid copepod *Eudiaptomus gracilis*. Microb. Ecol. 71, 835–844. doi: 10.1007/s00248-016-0734-8, PMID: 26888523 PMC4823325

[ref53] Reis G. C. D. de Carvalho G. H. A. Vilar M. C. P. Azevedo S. M. F. D. O. E. Ferrão-Filho A. D. S. (2023). Saxitoxin-producing *Raphidiopsis raciborskii* (Cyanobacteria) constrains *Daphnia* fitness and feeding rate despite high nutritious food availability. Toxics 11:693. doi: 10.3390/toxics11080693, PMID: 37624198 PMC10458173

[ref54] Ruiz T. Koussoroplis A. M. Danger M. Aguer J. P. Morel-Desrosiers N. Bec A. (2021). Quantifying the energetic cost of food quality constraints on resting metabolism to integrate nutritional and metabolic ecology. Ecol. Lett. 24, 2339–2349. doi: 10.1111/ele.13855, PMID: 34337842

[ref55] Rzymski P. Poniedziałek B. (2014). In search of environmental role of cylindrospermopsin: a review on global distribution and ecology of its producers. Water Res. 66, 320–337. doi: 10.1016/j.watres.2014.08.029, PMID: 25222334

[ref56] Sakai C. Ishida M. Ohba H. Yamashita H. Uchida H. Yoshizumi M.. (2017). Fish oil omega-3 polyunsaturated fatty acids attenuate oxidative stress-induced DNA damage in vascular endothelial cells. PLoS One 12:e0187934. doi: 10.1371/journal.pone.0187934, PMID: 29121093 PMC5679535

[ref57] Schälicke S. Sobisch L. Y. Martin-Creuzburg D. Wacker A. (2019). Food quantity-quality co-limitation: interactive effects of dietary carbon and essential lipid supply on population growth of a freshwater rotifer. Freshw. Biol. 64, 903–912. doi: 10.1111/fwb.13272

[ref58] Schoenberg S. A. Carlson R. E. (1984). Direct and indirect effects of zooplankton grazing on phytoplankton in a hypereutrophic lake. Oikos 42, 291–302. doi: 10.2307/3544397

[ref59] Schwarzenberger A. (2022). Negative effects of cyanotoxins and adaptative responses of *Daphnia*. Toxins 14:770. doi: 10.3390/TOXINS14110770, PMID: 36356020 PMC9694520

[ref60] Sikora A. Dawidowicz P. (2017). Breakage of cyanobacterial filaments by small- and large-sized *Daphnia*: are there any temperaturedependent differences? Hydrobiologia 798, 119–126. doi: 10.1007/s10750-015-2436-5

[ref61] Soares M. C. S. Rocha M. I. D. A. Marinho M. M. Azevedo S. M. Branco C. W. Huszar V. L. (2009). Changes in species composition during annual cyanobacterial dominance in a tropical reservoir: physical factors, nutrients and grazing effects. Aquat. Microb. Ecol. 57, 137–149. doi: 10.3354/ame01336

[ref62] Søndergaard M. Liboriussen L. Pedersen A. R. Jeppesen E. (2008). Lake restoration by fish removal: short-and long-term effects in 36 Danish lakes. Ecosystems 11, 1291–1305. doi: 10.1007/s10021-008-9193-5

[ref63] Stanley D. Kim Y. (2019). Insect prostaglandins and other eicosanoids: from molecular to physiological actions. Adv. Insect Physiol. 56, 283–343. doi: 10.1016/bs.aiip.2019.01.003

[ref64] Taipale S. J. Galloway A. W. Aalto S. L. Kahilainen K. K. Strandberg U. Kankaala P. (2016). Terrestrial carbohydrates support freshwater zooplankton during phytoplankton deficiency. Sci. Rep. 6:30897. doi: 10.1038/srep30897, PMID: 27510848 PMC4980614

[ref65] Tang Y. Su L. Xu R. Wang S. Su Y. Liu Z.. (2023). Response of zooplankton to inputs of terrestrial dissolved organic matter: food quality constraints induced by microbes. Limnol. Oceanogr. 68, 709–722. doi: 10.1002/lno.12304

[ref66] Tang Y. Yang X. Xu R. Zhang X. Liu Z. Zhang Y.. (2019). Heterotrophic microbes upgrade food value of a terrestrial carbon resource for *Daphnia magna*. Limnol. Oceanogr. 64, 474–482. doi: 10.1002/lno.11052

[ref67] Tang Y. Zhou D. Su L. Liu Z. Zhang X. Dumont H. J. (2021). *Vallisneria natans* detritus supports *Daphnia magna* somatic growth and reproduction under addition of periphyton. Aquat. Ecol. 55, 579–588. doi: 10.1007/S10452-021-09846-5

[ref68] Thomas P. K. Kunze C. Van de Waal D. B. Hillebrand H. Striebel M. (2022). Elemental and biochemical nutrient limitation of zooplankton: a meta-analysis. Ecol. Lett. 25, 2776–2792. doi: 10.1111/ele.14125, PMID: 36223425

[ref69] Twining C. W. Bernhardt J. R. Derry A. M. Hudson C. M. Ishikawa A. Kabeya N.. (2021). The evolutionary ecology of fatty-acid variation: implications for consumer adaptation and diversification. Ecol. Lett. 24, 1709–1731. doi: 10.1111/ELE.13771, PMID: 34114320

[ref70] Wacker A. Martin-Creuzburg D. (2007). Allocation of essential lipids in *Daphnia magna* during exposure to poor food quality. Funct. Ecol. 21, 738–747. doi: 10.1111/j.1365-2435.2007.01274.x

[ref71] Wenzel A. Vrede T. Jansson M. Bergström A. K. (2021). *Daphnia* performance on diets containing different combinations of high-quality algae, heterotrophic bacteria, and allochthonous particulate organic matter. Freshw. Biol. 66, 157–168. doi: 10.1111/fwb.13626

[ref72] Willis A. Adams M. P. Chuang A. W. Orr P. T. O’Brien K. R. Burford M. A. (2015). Constitutive toxin production under various nitrogen and phosphorus regimes of three ecotypes of *Cylindrospermopsis raciborskii* ((Wołoszyńska) Seenayya et Subba Raju). Harmful Algae 47, 27–34. doi: 10.1016/j.hal.2015.05.011

[ref73] Wilson A. E. Sarnelle O. Tillmanns A. R. (2006). Effects of cyanobacterial toxicity and morphology on the population growth of freshwater zooplankton: meta-analyses of laboratory experiments. Limnol. Oceanogr. 51, 1915–1924. doi: 10.4319/lo.2006.51.4.1915

[ref74] Wiltshire K. H. Boersma M. Möller A. Buhtz H. (2000). Extraction of pigments and fatty acids from the green alga *Scenedesmus obliquus* (Chlorophyceae). Aquat. Ecol. 34, 119–126. doi: 10.1023/A:1009911418606

[ref75] Wu Z. Yang S. Shi J. (2022). Overview of the distribution and adaptation of a bloomforming cyanobacterium *Raphidiopsis raciborskii*: integrating genomics, toxicity, and ecophysiology. J. Oceanol. Limnol. 40, 1774–1791. doi: 10.1007/S00343-022-2003-7

