# Supplementary material for: Chlorella pyrenoidosa mitigated the negative effect of cylindrospermopsin-producing and non-cylindrospermopsin-producing Raphidiopsis raciborskii on Daphnia magna as a dietary supplement
Source: Front Microbiol. 2023 Nov 16;14:1292277. doi: 10.3389/fmicb.2023.1292277 (PMC10687560; doi:10.3389/fmicb.2023.1292277)
Supplement: Supplementary file 1 [file Table_1.DOCX]

**Supplementary tables**

Table S1. Detailed information of statistics.

| **Fatty acids** |  |  | F | Sig. (2-tailed) | **Somatic growth rate at juvenile(/d)** |  |  | F | Sig. (2-tailed) |
| --- | --- | --- | --- | --- | --- | --- | --- | --- | --- |
|  |  |  |  |  |  |  |  |  |  |
| **C18:2ω6** | **CP** | **N8** | 0.0161 | 0.0029 |  | **CP** | **N8** | 0.1059 | ＜0.001 |
|  |  | **QDH7** | 15.1941 | 0.0080 |  |  | **QDH7** | 2.3105 | ＜0.001 |
|  |  | **CS506** | 8.6779 | 0.0052 |  |  | **CS506** | 0.4014 | ＜0.001 |
|  | **N8** | **QDH7** | 12.0642 | 0.1697 |  |  | **CP:N8=1:1** | 0.2820 | ＜0.001 |
|  |  | **CS506** | 6.8553 | 0.0586 |  |  | **CP:QDH7=1:1** | 0.3805 | 0.3043 |
|  | **QDH7** | **CS506** | 3.0897 | 0.0729 |  |  | **CP:CS506=1:1** | 7.4884 | 0.0771 |
|  |  |  |  |  |  |  |  |  |  |
| **C18:3ω3** | **CP** | **N8** | 3.9895 | 0.4607 |  | **N8** | **QDH7** | 2.5168 | ＜0.001 |
|  |  | **QDH7** | 7.6622 | 0.0357 |  |  | **CS506** | 1.0781 | ＜0.001 |
|  |  | **CS506** | 0.0835 | 0.5901 |  |  | **CP:N8=1:1** | 1.0259 | ＜0.001 |
|  | **N8** | **QDH7** | 11.0834 | 0.1764 |  |  | **CP:QDH7=1:1** | 1.2188 | ＜0.001 |
|  |  | **CS506** | 2.3182 | 0.6757 |  |  | **CP:CS506=1:1** | 11.5495 | ＜0.001 |
|  | **QDH7** | **CS506** | 3.4538 | 0.0370 |  |  |  |  |  |
|  |  |  |  |  |  | **QDH7** | **CS506** | 5.3933 | 0.0032 |
| **C18:3ω6** | **CP** | **N8** | 11.0465 | 0.1227 |  |  | **CP:N8=1:1** | 6.4191 | ＜0.001 |
|  |  | **QDH7** | 8.0900 | 0.0385 |  |  | **CP:QDH7=1:1** | 6.9384 | ＜0.001 |
|  |  | **CS506** | 4.6345 | 0.2983 |  |  | **CP:CS506=1:1** | 15.5504 | ＜0.001 |
|  | **QDH7** | **CS506** | 1.0375 | 0.0005 |  |  |  |  |  |
|  |  |  |  |  |  | **CS506** | **CP:N8=1:1** | 0.1818 | ＜0.001 |
| **C20:4ω6** | **CP** | **N8** | 3.6862 | 0.2502 |  |  | **CP:QDH7=1:1** | 0.0964 | ＜0.001 |
|  |  | **QDH7** | 4.1978 | 0.0015 |  |  | **CP:CS506=1:1** | 1.5584 | ＜0.001 |
|  |  | **CS506** | 3.4209 | 0.0011 |  |  |  |  |  |
|  | **N8** | **QDH7** | 2.7906 | 0.4407 |  | **CP:N8=1:1** | **CP:QDH7=1:1** | 0.0266 | ＜0.001 |
|  |  | **CS506** | 0.4710 | 0.0334 |  |  | **CP:CS506=1:1** | 7.1255 | ＜0.001 |
|  | **QDH7** | **CS506** | 2.0284 | 0.0102 |  |  |  |  |  |
|  |  |  |  |  |  | **CP:QDH7=1:1** | **CP:CS506=1:1** | 5.3970 | 0.0189 |

| **Net reproduction** | **CP** | **N8** | 130.8297 | ＜0.001 | **Population intrinsic increase rate** | **CP** | **N8** | 1.1340 | ＜0.001 |
| --- | --- | --- | --- | --- | --- | --- | --- | --- | --- |
|  |  | **CP:N8=1:1** | 0.4844 | ＜0.001 |  |  | **CP:N8=1:1** | 9.1885 | ＜0.001 |
|  |  | **CP:QDH7=1:1** | 29.1196 | 0.0012 |  |  | **CP:QDH7=1:1** | 3.1389 | ＜0.001 |
|  |  | **CP:CS506=1:1** | 0.0202 | 0.0004 |  |  | **CP:CS506=1:1** | 1.9043 | ＜0.001 |
|  |  |  |  |  |  |  |  |  |  |
|  | **N8** | **CP:N8=1:1** | 41.2797 | ＜0.001 |  | **N8** | **CP:N8=1:1** | 1.3387 | ＜0.001 |
|  |  | **CP:QDH7=1:1** | 88.0359 | ＜0.001 |  |  | **CP:QDH7=1:1** | 0.0677 | ＜0.001 |
|  |  | **CP:CS506=1:1** | 61.5125 | ＜0.001 |  |  | **CP:CS506=1:1** | 4.3175 | ＜0.001 |
|  |  |  |  |  |  |  |  |  |  |
|  | **CP:N8=1:1** | **CP:QDH7=1:1** | 19.4206 | 0.9859 |  | **CP:N8=1:1** | **CP:QDH7=1:1** | 0.9396 | ＜0.001 |
|  |  | **CP:CS506=1:1** | 0.2753 | 0.0282 |  |  | **CP:CS506=1:1** | 23.2752 | 0.0230 |
|  |  |  |  |  |  |  |  |  |  |
|  | **CP:QDH7=1:1** | **CP:CS506=1:1** | 25.5061 | 0.1668 |  | **CP:QDH7=1:1** | **CP:CS506=1:1** | 9.6401 | 0.0555 |

Table S2. Fatty acid composition of *C.pyrenoidosa* (CP) and three strains of *R.raciborskii*  (N8, QDH7 and CS506). Data were expressed as μg mg C^-1^ (n=3, mean (SD))

| Lipid | CP | N8 | QDH7 | CS506 |
| --- | --- | --- | --- | --- |
| C14:0 | 1.81(0.64) | 0.16(0.11) | 0.31(0.05) | 1.27(0.43) |
| C15:0 | 0.30(0.03) | 0.07(0.05) | 0.14(0.01) | 0.21(0.03) |
| C15:1ω5 | -- | -- | 0.06(0.00) | -- |
| C16:0 | 65.90(32.35) | 41.73(12.56) | 32.33(0.18) | 99.86(29.85) |
| C16:1ω5 | -- | 0.02(0.03) | -- | -- |
| C16:1ω7 | -- | 7.26(1.93) | 3.74(0.37) | 7.82(7.82) |
| C16:1ω9 | 1.95(0.46) | 0.03(0.05) | 0.07(0.01) | 5.74(4.95) |
| C16:1ω14 | -- | 0.05(0.04) | 0.03(0.03) | 0.15(0.02) |
| C16:2ω4 | -- | 0.17(0.12) | 0.19(0.00) | 0.56(0.56) |
| C16:2ω6 | 1.86(0.25) | 0.24(0.18) | 0.46(0.06) | 1.89(0.67) |
| C16:3ω3 | 2.17(0.28) | -- | -- | -- |
| C16:3ω6 | 0.16(0.03) | -- | -- | -- |
| C17:0 | 0.11(0.09) | -- | 0.03(0.03) | 0.04(0.04) |
| C18:0 | 20.13(13.21) | 0.61(0.50) | 1.71(1.20) | 8.43(3.49) |
| C18:1ω5 | 0.93(0.14) | 0.23(0.33) | 0.28(0.28) | -- |
| C18:1ω8 | -- | 0.23(0.33) | 0.02(0.02) | 0.05(0.05) |
| C18:2ω6 | 5.53(0.59) | 1.78(0.57) | 0.94(0.02) | 0.70(0.17) |
| C18:2ω8 | -- | -- | -- | 0.08(0.08) |
| C18:3ω3 | 11.68(1.43) | 14.00(3.78) | 8.52(0.16) | 12.66(2.32) |
| C18:3ω6 | 0.22(0.16) | -- | 0.76(0.03) | 0.36(0.06) |
| C18:4ω3 | 7.68(1.39) | -- | -- | -- |
| C20:0 | 0.09(0.04) | -- | -- | -- |
| C20:4ω6 | 0.76(0.12) | 3.40(2.77) | 5.09(0.54) | 10.67(2.04) |
| C22:0 | 0.27(0.06) | -- | -- | -- |
| C22:1ω9 | 0.19(0.08) | 0.02(0.03) | -- | 0.13(0.03) |
| C22:4ω7 | -- | 0.02(0.03) | 0.33(0.33) | 0.99(0.79) |
